# AlphaFold3: An Overview of Applications and Performance Insights

**DOI:** 10.3390/ijms26083671

**Published:** 2025-04-13

**Authors:** Marios G. Krokidis, Dimitrios E. Koumadorakis, Konstantinos Lazaros, Ouliana Ivantsik, Themis P. Exarchos, Aristidis G. Vrahatis, Sotiris Kotsiantis, Panagiotis Vlamos

**Affiliations:** 1 Bioinformatics and Human Electrophysiology Laboratory, Department of Informatics, Ionian University, 49100 Corfu, Greece; koumadorakis@fleming.gr (D.E.K.); lakonstant@ionio.gr (K.L.); lianaiv@outlook.com (O.I.); exarchos@ionio.gr (T.P.E.); aris.vrahatis@ionio.gr (A.G.V.); vlamos@ionio.gr (P.V.); 2 Department of Mathematics, University of Patras, 26504 Rio, Greece; kotsiantis@upatras.gr

**Keywords:** AlphaFold3, prediction accuracy, protein modeling, structural biology, protein–ligand interactions, deep learning

## Abstract

AlphaFold3, the latest release of AlphaFold developed by Google DeepMind and Isomorphic Labs, was designed to predict protein structures with remarkable accuracy. AlphaFold3 enhances our ability to model not only single protein structures but also complex biomolecular interactions, including protein–protein interactions, protein–ligand docking, and protein-nucleic acid complexes. Herein, we provide a detailed examination of AlphaFold3’s capabilities, emphasizing its applications across diverse biological fields and its effectiveness in complex biological systems. The strengths of the new AI model are also highlighted, including its ability to predict protein structures in dynamic systems, multi-chain assemblies, and complicated biomolecular complexes that were previously challenging to depict. We explore its role in advancing drug discovery, epitope prediction, and the study of disease-related mutations. Despite its significant improvements, the present review also addresses ongoing obstacles, particularly in modeling disordered regions, alternative protein folds, and multi-state conformations. The limitations and future directions of AlphaFold3 are discussed as well, with an emphasis on its potential integration with experimental techniques to further refine predictions. Lastly, the work underscores the transformative contribution of the new model to computational biology, providing new insights into molecular interactions and revolutionizing the fields of accelerated drug design and genomic research.

## 1. Introduction

The emergence of artificial intelligence (AI) in molecular biology has dramatically transformed the approach by which researchers forecast and comprehend the structure of proteins and their interaction with other molecules [1]. Among the revolutionary tools that have arisen, AlphaFold, the advanced machine-learning-based model developed by DeepMind, has set new standards for computational biology [2]. AlphaFold’s success in accurately predicting protein structures from amino acid sequences has revolutionized various fields, from drug discovery to disease understanding. In 2020, AlphaFold2 was the winner of the championship in the 14th Critical Assessment of Structure prediction (CASP14), presenting its neural network modules, evoformer and the structure module [3]. To delve into the intricacies, the output of AlphaFold2 consists of the 3D coordinates of all heavy atoms in the molecules, supplemented by a confidence score. However, it lacks the ability to predict critical elements of protein structures, as the model is unable to directly predict 3D structures solely from a raw sequence. Undoubtedly, deployment of predictive machine learning (ML) techniques such as the AlphaFold algorithm also marks a significant breakthrough in exploring protein-folding problems [4]. In 2024, DeepMind, in collaboration with Isomorphic Labs, introduced the revolutionary model. The release of AlphaFold3 has further expanded the capabilities of its predecessors, incorporating advanced AI techniques to handle a broader range of biomolecular challenges [5].

## 2. AlphaFold3 Improvements and Challenges

Unlike AlphaFold2, which relied on template-based methods [6], the advanced algorithm introduces a diffusion-based model, enhancing its ability to predict not only protein structures but also the intricate interactions between proteins, nucleic acids, and small molecules. As a result, AlphaFold3 holds the potential to accelerate research in areas such as drug design, systems biology, and molecular engineering, offering unprecedented accuracy in modeling complex biological systems. However, despite its promising capabilities, there are still challenges that need to be addressed, particularly in terms of predicting dynamic, flexible, and disordered regions within biomolecules. In this context, a comprehensive analysis of AlphaFold3 was presented, highlighting its advancements over previous versions [7]. AlphaFold3 significantly extends the predictive power of protein structures by incorporating a diffusion-based model, which is a key departure from AlphaFold2’s reliance on the structural module for amino acid framework predictions. This approach allows AlphaFold3 to handle a broader range of biomolecules, including proteins, nucleic acids, and small molecule complexes. This study emphasizes the application of AlphaFold3 in de novo biomolecule design, a novel feature enabled by the model’s ability to generate 3D structures directly from input sequences [7]. Furthermore, building on the predictive capabilities of AlphaFold3, the model’s expanded potential in predicting biomolecular interactions with unprecedented accuracy was explored [8]. Whereas AlphaFold2 was highly effective in predicting the structures of monomeric proteins, AlphaFold3 has extended this capability to more complex interactions, including those involving proteins, nucleic acids, and small molecules. This extension allows for more precise predictions of protein–ligand interactions, an area of significant importance for drug discovery. This study emphasizes how AlphaFold3 surpasses traditional methods, such as docking simulations, by accounting for conformational changes during ligand binding, thus offering a more accurate representation of binding affinities and pose configurations [8].

Likewise, the major improvements in AlphaFold3’s ability to predict biomolecular interactions are highlighted, particularly in protein–ligand and protein–protein interactions [5]. The authors assert that AlphaFold3’s performance surpasses earlier models in predicting not only individual protein structures but also more complex interactions between multiple biomolecules. This includes a more accurate prediction of binding sites and affinities, which are crucial for advancing drug development. The study demonstrates that AlphaFold3’s diffusion-based approach can predict protein–ligand interactions with remarkable precision, making it a highly valuable tool in both basic and applied molecular biology. However, the study acknowledges that the model’s ability to predict dynamic systems and disordered regions remains an area for enhancement, requiring ongoing research to fully capture the complexity of biomolecular interactions [5]. Complementing these advancements in protein–ligand predictions, a broader review of AI-driven techniques in protein structure prediction is provided, with particular attention to AlphaFold3 [9]. The evolution from traditional computational methods, such as homology modeling and threading, to state-of-the-art deep learning (DL) models, like AlphaFold and its successors, is observed. However, ongoing challenges, such as the difficulty in predicting protein dynamics, conformational changes, and interactions between biomolecules, are also addressed.

As AlphaFold3 continues to advance our understanding of protein structure and interactions, its impact on drug discovery is becoming increasingly apparent. Desai et al. provide a comprehensive study of AlphaFold3’s influence on drug design, focusing on how the model’s ability to predict protein–ligand interactions with high accuracy has revolutionized the way researchers approach drug development [10]. By leveraging DL techniques, the model can predict binding sites with greater precision than traditional methods, allowing for the rapid identification of potential drug targets. However, the authors also point out that challenges remain, particularly in predicting the dynamics and kinetics of protein–ligand interactions, which are crucial for the successful development of therapeutics. As AlphaFold3 continues to integrate into drug development pipelines, it is poised to reduce the time and cost traditionally associated with drug testing and validation, offering promising prospects for future therapeutic breakthroughs [10]. Moreover, Bargmann and Bohrer discuss the profound implications of AlphaFold3 for pharmaceutical patent law, particularly in the context of monoclonal antibody development [11]. The study suggests that AlphaFold3’s precision in predicting antibody–antigen interactions can help generate broader and more specific patent claims, leading to a new era of drug development. This shift in the way patents are granted will likely lead to reduced costs and faster timelines in drug discovery. Additionally, the paper emphasizes the broader implications of AI-driven protein structure prediction in biomedicine, highlighting how the advanced algorithm could redefine pharmaceutical companies’ approaches to intellectual property and drug development.

AlphaFold3’s ability was further explored to decode complex biomolecular behavior, particularly its role in enabling de novo biomolecule design [12]. By integrating a diffusion-based generative model, the algorithm has improved its capacity to model not only protein structures but also intricate biomolecular assemblies, such as protein–protein interactions, nucleic acids, and small molecule complexes. This shift has significant implications for drug discovery, where AlphaFold3’s predictive power can guide the development of novel therapeutic agents. However, the authors also caution that dynamic and disordered regions of proteins remain a challenge, underscoring the need for future advancements to fully capture the structural diversity and flexibility of biomolecules [12]. In addition, a scientometric analysis that underscores the rapid expansion of research on AlphaFold was given, highlighting its transformative role in structural biology and drug discovery [13]. The study reveals a staggering 180.13% annual growth in AlphaFold-related research, emphasizing its rising significance in the scientific community. However, while AlphaFold has made tremendous strides, persistent gaps have been noted, particularly in areas like protein–ligand interactions where AlphaFold3 shows promise but also faces challenges [13].

Expanding on the intersection of AI and molecular modeling, Zhou and Huang provide a deep dive into how AlphaFold3 has revolutionized enzyme design [14]. While AlphaFold3 excels at modeling static protein structures, the study acknowledges the challenges posed by dynamic systems, such as enzymes with flexible active sites. To address these issues, the importance of combining AlphaFold3’s predictions with molecular dynamics simulations was emphasized, enabling more accurate representations of enzyme behavior in complex biological contexts. The authors argue that the model’s predictive power, integrated with ML, can catalyze breakthroughs in biocatalysis processes and therapeutic enzyme development, showcasing the vast potential of AI in enzyme engineering [14].

Similarly, the broader impact of DL techniques, including AlphaFold3, on the landscape of biomolecular structure prediction was explored [15]. A comparison of AlphaFold3 with other deep learning models was performed, underscoring its ability to predict not only protein structures but also more intricate biomolecular systems such as protein–RNA and protein–ligand complexes. The authors particularly note the algorithm’s improvements in predicting multi-chain protein assemblies and protein–protein interactions, areas where previous models struggled; however, they acknowledge that predictions of disordered proteins still need refinement [15].

Building on the concept of using AI in complex biological systems, Zonta and Pantano examine how AlphaFold3 can be applied to the field of mechanobiology [16]. Their work focuses on how protein structures influence cellular mechanics and force sensing—critical aspects of tissue function and response to mechanical stress. By applying AlphaFold3 to model integrin-binding proteins, cytoskeletal proteins, and membrane-associated proteins, the authors provide valuable insights into cellular responses to mechanical stress. Additionally, Chakravarty et al. focus on a crucial limitation of AlphaFold3 in predicting alternative protein folds—an issue where the model often struggles to capture the diverse range of conformations that proteins may adopt [17]. While AlphaFold3 excels in predicting the most stable conformation of a protein, it faces challenges when proteins exhibit fold-switching behavior or alternative structural states. The study provides specific examples, such as BCCIPa and DZZB, where AlphaFold3’s predictions were built upon training set biases, leading to discrepancies in the modeling of shape-changing metamorphic proteins in response to cellular conditions. The study implies that future iterations of AlphaFold should incorporate strategies to better predict alternative folding, such as improved multiple sequence alignment (MSA) modifications and multi-state modeling [17]. Moving to a broader perspective on AlphaFold3’s applications, the potential of the new algorithm in studying complex biomolecular assemblies, particularly metabolons, was depicted [18]. Both AlphaFold 2 and AlphaFold 3 have been used to model the pyruvate dehydrogenase complex (PDHc) by predicting the structures of its enzymatic components and their interactions, with the new model accurately predicting transient interactions within the complex, requiring human supervision and alignment with biochemical insights for enhanced outcomes. Lastly, the importance of combining AlphaFold3’s predictions with experimental techniques like cross-linking and cryogenic electron microscopy (cryo-EM) was stressed to improve model accuracy.

The debate surrounding the open-source availability of AlphaFold3 is also addressed in a recent study exploring the ongoing controversy surrounding the model’s restricted access [19]. Callaway discusses the challenges of using AlphaFold3 as a black-box tool, with limited access to its internal workings and restrictions on commercial use. This has led to efforts by researchers, including Mohammed AlQuraishi and David Baker, to develop open-source alternatives and retrain the model with proprietary data. These initiatives aim to enhance AlphaFold3’s performance, particularly in the context of drug discovery and biomolecular modeling. The author concludes that real progress in the field of computational biology will depend on greater collaboration and transparency, ensuring that AI tools like AlphaFold3 are used responsibly and effectively [19]. Furthermore, other open-source DL models, such as Boltz-1, achieve AlphaFold3-level accuracy in predicting the 3D structures of biomolecular complexes [20].

The findings discussed herein reflect the transformative impact of AlphaFold3 on biochemistry, structural biology, and drug design. Each study highlights different aspects of the model’s capabilities, from predicting complex protein structures to advancing the understanding of biomolecular interactions and dynamics. As these works collectively illustrate, AlphaFold3 has not only addressed long-standing challenges in molecular biology but has also paved the way for new research opportunities and practical applications. The following sections delve further into these advances, exploring the specific contributions and implications of the new AI model in diverse biological and therapeutic contexts. The first one focuses on its transformative impact on structural biology, examining how this tool has been utilized in the study of protein–protein interactions, protein–nucleic acid interactions, and other dynamic biomolecular complexes. The second section directs emphasis toward drug discovery, demonstrating how the algorithm assists in identifying drug targets, drug–ligand binding, and the design of new therapeutics.

## 3. Transformative Insights into Biomolecular Structures and Modeling

AlphaFold3 marks a significant breakthrough in computational structural biology, enhancing the accuracy and scope of protein structure predictions. While the previous version of the model transformed protein structure prediction, AlphaFold3 integrates a more refined generative model capable of predicting a broader range of biomolecular interactions, introducing important simplifications to the model’s architecture. This includes not only proteins but also nucleic acids, ligands, and complex multi-subunit assemblies, which allow researchers to explore dynamic biomolecular processes with greater precision, emphasizing the role of 20 DL networks and algorithmic optimization for model training, validation, and evaluation [21]. One of the most impressive features of AlphaFold3 is its ability to predict protein–ligand interactions, an area traditionally challenging for computational models. This advancement has profound implications for drug discovery, enabling the identification of novel targets and the design of more effective therapeutics. Additionally, AlphaFold3’s ability to model multi-protein complexes and dynamic biomolecular interactions provides valuable insights into cellular processes, protein-folding mechanisms, and the molecular basis of diseases [5].

AlphaFold3’s application to structural biology extends beyond protein structure prediction. It is proving to be a vital tool in elucidating the roles of proteins in complex biological systems. Its integration with experimental data, such as cryo-electron microscopy (cryo-EM) and molecular dynamics simulations, further strengthens its capabilities, making it a comprehensive resource for understanding the functional architecture of proteins and their interactions within living systems [22]. The evaluation of AlphaFold3 in docking fatty acids to human fatty acid-binding proteins (FABPs) sheds light on its broader potential to predict ligand–protein interactions [23]. AlphaFold3’s ability was tested to predict binding sites and poses for various fatty acids across a dataset of human FABPs, and the outcomes were compared to experimental structures from the Protein Data Bank (PDB), focusing on binding accuracy, root-mean-square deviations (RMSD), and interaction energy calculations. While AlphaFold3 demonstrated high accuracy in identifying primary binding sites for saturated fatty acids, its performance declined for unsaturated and branched-chain variants, reflecting challenges in modeling flexible or unconventional ligands. This study underscores AlphaFold3’s growing role in ligand–protein interaction modeling, particularly for standard binding scenarios [23]. The study by You et al. [24] explored the integration of the new model with gold nanoparticle-based lateral flow immunoassays (LFIAs) for the rapid detection of pyraclostrobin, a commonly used agricultural fungicide. The results demonstrated that AlphaFold3 significantly contributed to improving antibody design, leading to enhanced detection limits for pyraclostrobin compared to conventional methods, demonstrating innovative application of the algorithm in environmental monitoring and offering a robust, rapid, and scalable approach to detect agricultural residues with high accuracy.

AlphaFold3 was further utilized to investigate its ability to predict the 3D structures of large RNA molecules, focusing on assessing its scalability and accuracy [19,25]. The study applied AlphaFold3 to a dataset of 200 RNA molecules ranging from small hairpins to large ribozymes, and the model indicated confidence scores (pLDDT) to correlate model reliability with structural complexity. The findings revealed that AlphaFold3 performed adequately for small to medium-sized RNAs, producing accurate models with RMSD values comparable to those of specialized RNA modeling tools. However, its accuracy declined significantly for larger RNAs with complex tertiary interactions, highlighting the limitations of its protein-centric architecture in handling RNA-specific features. Moreover, AlphaFold3 has been applied to rare but functionally significant phenomena, such as the modeling of knotted proteins [26]. The model accurately predicted the occurrence of knots in well-characterized proteins, aligning closely with experimental data; however, potential undiscovered complexity in protein folding has been highlighted. These findings underline AlphaFold3’s potential as a powerful tool for studying protein topology and provide insights into the role of knots in protein function, stability, and evolution [26].

Further showcasing its versatility, AlphaFold3 has bridged the gap between sequence- and structure-based protein classifications. A recent study aimed to integrate sequence-based (Pfam) and structure-based (ECOD) classifications to improve the annotation of uncharacterized protein families, leveraging the advanced model as a central tool for structural predictions [27]. AlphaFold3 was applied to model a dataset of 1200 protein domains, specifically those lacking experimentally resolved structures, and the findings showed reliably predicted domain structures were observed, enabling links between ECOD and Pfam for many previously uncharacterized families. Moreover, Tian et al. explored the structural basis of Zn^2+^-mediated non-competitive inhibition of caspase-3, a critical enzyme in apoptosis, integrating AlphaFold3’s predictive capabilities with molecular docking and dynamic simulations to identify Zn^2+^-binding sites and their impact on enzyme activity [28]. The study accurately demonstrated key structural feature prediction of caspase-3, facilitating the identification of Zn^2+^-binding sites that disrupt active site geometry through allosteric effects and paving the way for therapeutic strategies targeting metal ion modulation in apoptosis-related diseases.

Exploration of the impact of benzimidazole resistance-associated mutations on the dimerization of hookworm tubulin was employed, assessing whether enhanced tubulin–tubulin interactions represent an additional resistance mechanism [29]. Molecular dynamics simulations were further performed on AlphaFold3’s models to assess the dynamic stability of the predicted dimers and resistance-associated mutations, and improved tubulin dimerization by stabilizing the interfacial interactions between *α*- and *β*-tubulin subunits was detected. These stabilizing effects likely offset the destabilizing influence of benzimidazole binding and may provide an alternative resistance mechanism, highlighting the role of AlphaFold3 in uncovering molecular mechanisms underlying drug resistance as well as offering insights for developing new strategies to overcome resistance in parasitic helminths. Furthermore, in autophagy research, a fragment-based screening approach was developed to enhance AlphaFold3’s ability to identify LC3-interacting regions (LIRs) within proteins, addressing the limitations of full-length protein predictions in identifying both canonical and noncanonical LIR motifs [30]. Validation was carried out using cryo-electron microscopy and biochemical assays to confirm the binding poses and functional relevance of predicted interactions, uncovering potent interaction mechanisms that may inform future therapeutic developments targeting autophagic pathways. In addition, Coskuner-Weber used AlphaFold3 to model *α*-synuclein, a key protein involved in neurodegenerative diseases, predicting regions of transient secondary structure in *α*-synuclein, which served as starting points for replica exchange molecular dynamics (REMD) simulations [31]. The outcomes produced conformational ensembles that aligned with experimental NMR data, illustrating the dynamic nature of IDPs.

In a recent work on SARS-CoV-2 main protease (M_pro_), Aniana et al. used AlphaFold3 to predict protein–protein interaction interfaces and investigate alternate dimerization and higher-order assemblies [32]. Molecular dynamics simulations also assessed the stability and flexibility of the assemblies over time, and the predictions were validated through cryo-electron microscopy and biochemical assays, uncovering unique interfacial configurations that correlated with changes in enzymatic activity and uncovering noncanonical interactions in viral proteases. AlphaFold3 has also been employed to investigate the structural regulation of Polo-like kinases (PLKs), essential enzymes in cell division and cancer progression, and their conformational changes associated with phosphorylation and regulatory binding, revealing critical residues that modulate protein activation and provide insights into their role in cancer progression [33]. AlphaFold3 has also been instrumental in understanding plant innate immunity mechanisms to model the assembly and activation processes of NLR proteins. The models included detailed structural insights into inter-domain interactions, ATP-binding motifs, and oligomerization interfaces, successfully predicting key aspects of the hexameric resistosome structure, including the precise inter-domain arrangements and conformational shifts required for activation [34]. By combining computational predictions with experimental validation, this research advances the understanding of plant immune mechanisms and offers valuable insights for engineering disease-resistant crops. In another study, the structural and dynamic basis of anomalous fluorescence emissions in single *α*-helical peptides in solution was explored, combining computational modeling and experimental validation to uncover the mechanisms behind their unique optical properties [35]. The findings showed that AlphaFold3 accurately predicted critical structural motifs, such as helical turns and hydrophobic core packing, that influence fluorescence behavior, while residue-specific interactions, including side-chain stacking and hydrogen-bonding networks, were identified as major contributors to fluorescence efficiency, paving the way for designing biomolecules with tunable optical properties, advancing applications in bioimaging and optoelectronics.

In the context of exploring structural diversity in protein systems, a comprehensive characterization of cysimiditides, a unique subclass of ribosomally synthesized and post-translationally modified peptides (RiPPs), was provided [36]. AlphaFold3 was applied to predict the tertiary structures of multiple cysimiditides, focusing on the spatial arrangement of cysteine residues within the Zn-binding motif, revealing intricate hairpin conformations stabilized by zinc coordination. The study also explored interactions between cysimiditides and methyltransferases involved in aspartimidylation, using molecular docking simulations based on AlphaFold3-generated models, providing critical insights into the structural and functional roles of the Zn-tetracysteine motif in stabilizing the hairpin architecture and facilitating enzymatic modifications. Additionally, AlphaFold3 has been used to elucidate the structural features of human F-ATP synthase, a target for mitochondrial diseases and cancer therapy [37]. The predictions aligned well with experimental data, identifying key binding sites on the F1 complex where small molecules could interfere with ATP production, highlighting the utility of the new model in drug discovery for developing therapies targeting F-ATP synthase to combat a variety of diseases [37].

Within the scope of cancer immunotherapy, the role of human T cell receptors (TCRs) in recognizing NRAS cancer neoantigens with AlphaFold3’s predictions was validated using experimental techniques such as peptide–MHC tetramer staining and TCR engagement assays to confirm the structural accuracy and binding specificity [38]. The new algorithm was also used to predict structural features of the GluN3 NMDA receptor, a protein involved in synaptic plasticity and various neurological disorders, focusing on its ligand-binding domains and ion channel architecture [39]. The models were analyzed for the positioning of key residues that interact with neurotransmitters and other regulatory proteins, and the predicted structures were compared with existing cryo-EM data to assess the accuracy of the predictions, showing a strong representation of the receptor’s structure, particularly in the ligand-binding domains. Building on AlphaFold3’s comparative structural modeling capabilities, heme-binding sites in heme proteins were analyzed by comparing AlphaFold3’s predictions to those of its predecessor, AlphaFold2 [40]. The study evaluated whether AlphaFold3 offered improvements in accurately modeling the coordination of the heme group and its interactions with surrounding protein residues, indicating that the advanced model significantly outperformed the previous one, particularly for proteins with complex or flexible heme-binding pockets. In another therapeutic application, Li et al. examined the impact of N-terminal capping on the stability and selectivity of GeX-2, an analogue of the *α*Oconotoxin, toward the human *α*_9_*α*_10_ nicotinic acetylcholine receptor [41]. The analysis showed that N-terminal capping significantly improved the serum stability and selectivity of GeX-2, providing insights into the peptide’s potential for pain management and other therapeutic applications, highlighting the utility of AlphaFold3 in guiding the design of peptides with improved pharmacokinetic properties and receptor specificity. Moreover, the epitopes of the fused ESAT6/Tb10.4 protein, implicated in the immune response to Mycobacterium tuberculosis, were analyzed, and the results showed that the recombinant protein induced a robust immune response, validating the computational predictions made by AlphaFold3 [42].

AlphaFold3’s comparative abilities were also explored in a recent study of the Rad52 superfamily, which plays an essential role in DNA repair and genome stability [43]. Experimental validations, including crystallographic structures and mutagenesis studies, confirmed the accuracy of computational predictions, providing critical insights into the molecular mechanisms of genome maintenance and emphasizing the potential for targeting these pathways in cancer therapy. In another study, an exploration of the integration of Mendelian randomization with AlphaFold3 predictions was executed to identify *β*-protein biomarkers in Alzheimer’s disease [44]. The authors combined genetic data with protein structural predictions to investigate how mutations influence *β*-protein misfolding and aggregation, a hallmark of Alzheimer’s pathology, indicating that the new model could effectively connect genetic variation to structural disruption, advancing the understanding of Alzheimer’s disease mechanisms. LuxSAI-2, a system facilitating the dissemination of antibiotic-resistant plasmids in Klebsiella pneumoniae, was recently explored, examining how the LuxS enzyme and the AI-2 signaling pathway contribute to horizontal gene transfer, a key mechanism in antibiotic resistance [45]. The models which were derived through AlphaFold3 were further validated with molecular dynamics simulations and experimental conjugation assays, revealing critical binding sites mediating plasmid transfer. The findings, providing new insights into bacterial communication and resistance mechanisms, demonstrate the potential of the new AI model to guide the development of antimicrobial therapeutic strategies [45]. Kovalenko et al. compared predicted structures of the plastocyanin–cytochrome f complex with those obtained through cryo-EM and NMR methods [46]. AlphaFold3 successfully modeled the interaction interfaces of and dynamic conformational changes in the complex, with predictions validated against cryo-EM reconstructions, demonstrating its utility in studying electron transport systems and its compatibility with experimental techniques for understanding dynamic protein interactions [46].

Wee and Wei (2023) employed AlphaFold3 to predict protein–protein binding free energy changes upon mutation, using the SKEMPI 2.0 database [47]. The authors combined the model’s predictions with a topological DL model, MT-TopLap, showing predictions aligned closely with experimental results, emphasizing its role in modeling protein interactions, particularly in stable complexes, while identifying challenges in predicting structural changes for flexible domains. In another work, the performance of AlphaFold3, along with AlphaFold2 and ESMFold3, was explored in predicting the structures of chimeric proteins—artificially fused proteins combining sequences from distinct sources [48]. AlphaFold3, despite its advancements in structure prediction using a diffusion-based approach, was found to exhibit significant limitations in accurately predicting the structures of terminal tags when placed in the context of a chimeric protein. While AlphaFold3 demonstrated strong predictive capabilities for isolated protein components, its reliance on multiple sequence alignment (MSA) was less effective in the chimeric setting, as the chimeric sequences disrupt the evolutionary signals leveraged by MSAs. However, while AlphaFold3’s performance was examined for selection cases due to limited accessibility, the authors concluded that significant improvements are needed to make the algorithm more reliable for predicting chimeric protein structures. This underscores the importance of developing complementary tools or refinements to the new model for handling complex synthetic protein scenarios [48]. Lastly, Mano et al. applied AlphaFold3 to model the ATP-binding domains and hexameric assembly of the MoxR AAA+ ATPase from *Synechococcus* sp. strain NKBG15041c, a protein involved in stress resistance, revealing functional regions responsible for ATP hydrolysis and stress adaptation [49].

Table 1 summarizes the studies reviewed in this section, underscoring AlphaFold3’s transformative impact on structural biology and understanding of molecular mechanisms. From accurately modeling protein–ligand interactions and predicting RNA structures to elucidating protein–protein complexes and their dynamics, AlphaFold3 has pushed the boundaries of what computational tools can achieve. Its integration with experimental techniques such as cryo-electron microscopy, molecular docking, and molecular dynamics simulations has further solidified its position as a cornerstone of modern structural biology. Despite these advancements, certain challenges persist. AlphaFold3’s performance in modeling highly flexible proteins, RNA-specific interactions, and complex ligand binding scenarios highlights areas where further refinement is needed [50]. The combination of AlphaFold3 with complementary computational tools and experimental validation has proven effective in addressing these limitations, emphasizing the importance of interdisciplinary approaches in tackling unresolved questions in molecular biology and protein engineering.

## 4. Applications and Advancements of AlphaFold3 in Biomolecular Interactions

The present review provides a comprehensive examination of AlphaFold3’s transformative contributions in predicting single protein structures, showcasing its applications across diverse biological systems and highlighting its role in addressing some of the most challenging problems in protein complexes and biomolecular interactions. On the other hand, critical limitations and areas for improvement are depicted, particularly in modeling flexible structures, dynamic interfaces, and highly complex biological assemblies. Building on this premise, the next section delves deeper into the comparative performance of AlphaFold3 in specific, emerging areas of computational biology. These include its application to nanobody epitope prediction, fold-switching conformations, RNA and metal-binding interactions, and specialized challenges like PROTAC-mediated interfaces and peptide–MHC binding [51,52,53,54,55,56,57,58,59,60,61,62,63,64]. By analyzing the model’s strengths, limitations, and evolution from prior iterations, this section tries to explore the broader implications of AI-driven methods in tackling specialized tasks and advancing our understanding of biomolecular systems. As we turn to these frontier applications, the focus shifts toward benchmarking AlphaFold3 against other computational approaches, investigating its reliability, and assessing its integration with experimental validation. The studies herein offer critical insights into how the algorithm is shaping—and being shaped by—the growing demands of computational structural biology, expanding the limits of what AI-based models can accomplish.

AlphaFold3 has been instrumental in advancing the study of nanobody–antigen and antibody–antigen interactions, which are critical for therapeutic and diagnostic applications. Eshak et al. critically evaluated AlphaFold3 alongside AlphaFold2-Multimer for their ability to predict nanobody epitopes [51]. Using a dataset of 70 nanobody–antigen complexes, AlphaFold3 demonstrated an improved epitope identification success rate (47.1% compared to 32.8%), highlighting key factors influencing prediction accuracy, such as CDR3 loop length, conformation, and residue composition. In another study, Hitawala and Gray explored the model’s docking capabilities using a benchmark dataset of 150 antibody and nanobody complexes [52], with accurate prediction of standard antibody–antigen interfaces performed with high ipTM scores correlating to low RMSD values for many complexes. As we shift focus, AlphaFold3’s applications in RNA and metal-binding interactions emerge as another vital area of exploration. These studies reveal its ability to model diverse biomolecular systems, highlighting both its potential and its limitations in handling non-protein-centric challenges. AlphaFold3 was employed to predict RNA–ligand complexes, benchmarking its performance against other RNA 3D structure prediction tools, and it excelled in modeling binding interfaces and identifying biologically accurate interaction sites, outperforming several tested methods [53].

Bernard et al. applied AlphaFold3 to a dataset of 150 RNA structures, ranging from simple hairpins to complex ribozymes, to further evaluate its RNA modeling capabilities [54]. While the model performed well for secondary structures, its predictions diverged significantly for tertiary configurations, often producing over-simplified or incorrect folds for larger RNAs with intricate base-pairing networks. In addition, Dürr and Rothlisberger evaluated AlphaFold3’s performance in predicting metal-binding sites, an area traditionally challenging for computational tools due to the complex electronic properties of metal ions [55]. Using a dataset of 887 proteins containing biologically relevant metals such as zinc, calcium, magnesium, and iron, the authors benchmarked the new model against specialized tools like Metal3D and AllMetal3D, demonstrating high confidence in metal ion placement for physiologically relevant binding sites, particularly those with three or more coordinating residues. The study also highlighted its adaptability to mutations in metal-binding residues, which contributed to its success in modeling certain metalloproteins. However, challenges persisted in predicting novel metal-binding motifs and multi-metal systems, with AlphaFold3 struggling to generalize its predictions for stoichiometry and unique coordination environments [55].

AlphaFold3’s application to repetitive or unconventional protein structures has provided new insights into its strengths and limitations, particularly when handling synthetic and highly repetitive sequences. Pratt et al. analyzed AlphaFold2’s tendency to produce biologically implausible *β*-solenoid structures for synthetic repeat proteins [56]. Using a dataset of perfect repeat sequences with varying lengths and secondary structure propensities, the study revealed that AlphaFold2 frequently predicted high-confidence but unrealistic *β*-solenoid configurations. In contrast, AlphaFold3 mitigated these biases, producing fewer *β*-solenoid predictions and assigning lower confidence scores to improbable structures [56]. AlphaFold3’s capacity to model proteins with highly dynamic structural states, such as fold-switching proteins, provides another perspective on its ability to handle structural variability. Chakravarty et al. assessed the model’s ability to predict fold-switched conformations, focusing on proteins capable of adopting two distinct structural states [57]. Using a dataset of 92 fold-switching proteins, they generated over 280,000 structural predictions under various conditions, such as with and without evolutionary data, and the model successfully predicted only 7 fold-switching conformations, with accurate predictions often attributed to structural memorization from training data rather than generalized protein-folding principles [57]. Furthermore, Alkhouri et al. tested AlphaFold3’s robustness by introducing adversarial protein sequences designed to disrupt native folding patterns, and the model often retained high-confidence scores for adversarial sequences despite significant deviations in predicted structures, revealing vulnerabilities in its reliance on learned patterns rather than physical plausibility [58]. This study highlights a critical limitation of AI-driven protein modeling tools for applications in protein engineering and de novo sequence design, where robust predictions are essential.

AlphaFold3 has also demonstrated its potential in advancing therapeutic and experimental applications, particularly in modeling complex biomolecular interactions and guiding drug development efforts. A comparison of AlphaFold3 to its predecessor in modeling 276 human–parasite interactions across 15 parasitic species produced lower confidence scores, reflecting a more cautious approach to structural prediction [59]. DockQ scores revealed limited structural agreement between the predictions of AF2 and AF3, emphasizing their distinct approaches to modeling interaction dynamics. Lu et al. investigated AlphaFold3’s ability to predict mutational effects on protein–protein interactions using the SKEMPI dataset, which contains experimentally measured changes in binding energy (∆∆*G*) for 475 mutants across 42 protein complexes [60]. By calculating the ranking score difference between wild-type and mutant proteins, AF3 demonstrated smoother energy landscapes compared to traditional methods, providing unique insights into global mutational effects. Ensemble modeling approaches that integrated AlphaFold3 predictions with baseline methods significantly improved performance, underscoring its utility in studying protein–protein interactions and engineering therapeutic proteins.

Pereira et al. evaluated AlphaFold3’s potential in modeling PROTAC-mediated protein–protein interfaces, a critical aspect of targeted protein degradation therapies [61]. Using a dataset of 28 PROTAC-mediated complexes, the advanced model generated structural predictions that included accessory protein sequences to reduce search space and mimic physiological conditions. However, most predictions failed to meet acceptable DockQ quality thresholds, even with these modifications, underscoring the critical need for ligand data integration in modeling workflows to accurately capture PROTAC-mediated interactions. Moreover, Gomes et al. integrated AlphaFold3 with dynamic network analysis to enhance epitope prediction for PD-L1:Affibody interactions [62]. AlphaFold3 was used to generate structural models of the PD-L1:Affibody complex, which were further analyzed using dynamic network approaches to identify residues critical for binding stability and specificity. This integration significantly improved the precision of epitope predictions, with computational results aligning closely with experimentally validated sites. Lastly, AlphaFold3 was performed against sequence-based methods for predicting peptide–MHC class II (pMHC-II) binding cores, showing a diminution to its accuracy for less common alleles or peptides with unconventional binding motifs [63]. The synergistic use of AlphaFold3 with other methodologies, such as molecular dynamics simulations, dynamic network analysis, and sequence-based tools, points to an exciting future for computational structural biology, as Table 2 indicates. These integrations exemplify how AlphaFold3 can complement experimental data to provide deeper insights into biomolecular mechanisms.

## 5. Future Perspectives in AI-Driven Structural Predictions

AlphaFold3 has significantly advanced the field of structural biology, demonstrating its versatility across diverse applications. From protein–ligand interactions to RNA modeling, its predictive power has reshaped how researchers approach molecular studies. However, this progress comes with challenges that underline the complexity of biomolecular systems and the need for continuous development. In protein interaction modeling, AlphaFold3 has enabled more accurate predictions of standard interfaces, contributing to drug design and fundamental research [10]. Yet, its limitations in capturing noncanonical binding modes, dynamic protein states, and structurally unconventional targets reveal gaps in its ability to generalize across diverse biological systems. Addressing these gaps requires enhancements in both training datasets and algorithmic flexibility to better reflect the variability inherent in biological molecules.

Non-protein systems, such as RNA and metalloproteins, provide additional challenges for AlphaFold3 [50,64]. Its performance in simple RNA–ligand interactions and biologically relevant metal-binding sites is promising, but the tool struggles with more complex RNA tertiary folds and novel metal-binding motifs. Dynamic biomolecular systems, including fold-switching proteins and perturbation-resilient designs, test AlphaFold3’s ability to predict non-equilibrium states. Recent studies show that although AlphaFold3 performs well in predicting static structures, its accuracy diminishes when faced with highly flexible systems or sequences specifically designed to challenge predictive algorithms [17]. This underscores the need for integrating sampling techniques and energy-based models to improve its robustness in modeling biomolecular dynamics.

Complementary tools have emerged as critical extensions to AlphaFold3’s capabilities, addressing specific limitations and enabling broader applications, as presented in Table 3. AlphaBridge [65] provides a streamlined framework for analyzing macromolecular complexes, leveraging AlphaFold3’s confidence metrics for efficient validation and visualization. The AlphaBridge tool uses these metrics in combination with a graph-based community clustering approach to visualize binary interactions between predicted proteins. The resulting interaction maps, represented in chord diagrams, provide a comprehensive visual summary of the predicted interfaces and their associated confidence scores. This method allows researchers to easily identify likely interaction sites, rank them, and assess the reliability of the predictions before conducting further experimental work. By combining structure-based validation with a user-friendly interface, it offers a quick, reliable, and accessible way to analyze protein complexes [65]. Similarly, AlphaCRV [66] combines sequence- and structure-based clustering to enhance the reliability of protein–protein interaction predictions, particularly in large-scale proteome-wide analyses. Using AlphaFold3 models as input, AlphaCRV ranks potential protein–protein interactions based on structural and sequence-based clustering, identifying biologically relevant binders by their consistency in structure and topology. The pipeline filters out false positives by ensuring that interactions cluster together with low RMSD values.

Toward drug discovery, GalaxySagittarius-AF [67] has demonstrated the potential of combining AlphaFold3 with ligand-based approaches to identify novel drug targets across the human proteome. The key innovation of GalaxySagittarius-AF is its integration of predicted AlphaFold3 models, which augment the database with structures for proteins that lack experimental data. The system leverages a structure-based approach to search for potential drug targets by matching drug-like compounds to predicted protein structures. The database, now expanded to cover over 71,000 human protein structures, includes predicted binding sites and ligands, enabling the identification of potential targets for drug repurposing or new drug discovery. This synergy highlights how AlphaFold3’s structural predictions can be effectively integrated into workflows that address pressing challenges in therapeutic development [67]. Tools like AlphaFLOW-Lit further exemplify this trend by enabling efficient ensemble generation, essential for capturing protein flexibility and guiding drug design [68]. AlphaFLOW-Lit is a more efficient version of AlphaFLOW designed to accelerate the generation of protein ensembles. By conditioning the model on feature-specific data rather than using the full sequence information, AlphaFLOW-Lit reduces the computational burden by approximately 47 times compared to its predecessor, representing a significant advancement in protein dynamics simulation. Despite these advancements, AlphaFold3’s reliance on learned patterns sometimes results in overconfident predictions that lack physical plausibility. This issue becomes particularly evident in flexible or unconventional systems, where AlphaFold3 can fail to account for the full complexity of molecular interactions, as well as exploring RNA and RNA–protein complexes [5,69]. Tackling these challenges will require a combination of algorithmic refinements, enhanced training datasets, and hybrid workflows that integrate experimental data and advanced computational methodologies. AlphaFold3 has built the framework for groundbreaking work in structural biology; however, the next phase of development will hinge on overcoming these limitations. Exploiting its existing capabilities and combining them with complementary tools, users can fully leverage its potential to resolve increasingly complex biological issues.

## 6. Conclusions

The AlphaFold3 model, released in 2024, represents a transformative milestone in structural biology, predicting the three-dimensional structures of proteins and understanding biomolecular interactions and functions. Its unprecedented accuracy and versatility have expanded the scope of computational biology, enabling breakthroughs in areas ranging from protein–protein interactions to RNA modeling, metalloprotein studies, and dynamic biomolecular systems. The model’s integration into immunotherapy, drug discovery, and systems biology has demonstrated its value in addressing long-standing challenges. Its success in predicting protein interactions, as well as protein–ligand and protein–protein interfaces, underscores its utility for therapeutic applications. For example, AlphaFold3 was used for predicting protein–protein interaction interfaces such as SARS-CoV-2 M_pro_ dimerization, structural effects of N-terminal modifications such as N-terminal capping on GeX-2 stability, coordination of heme and protein residues, mutation effects on protein folding, peptide-MHC class II binding cores, and conformational changes such as plastocyanincytochrome f interactions. Similarly, advancements in understanding RNA structures and metal-binding proteins have highlighted its adaptability to non-protein systems, despite persistent limitations in these areas. While AlphaFold3 has addressed many complexities inherent to structural prediction, its reliance on pattern recognition over physical principles has exposed vulnerabilities, particularly in dynamic and unconventional systems. The inability to consistently predict fold-switching proteins, conversely disordered sequences, or RNA tertiary structures indicates the necessity for specialized adaptations and improved input data. These limitations, while significant, represent opportunities for the next generation of AI-driven models to incorporate energetics-based and sampling methodologies.

Looking ahead, the evolution of AlphaFold3 and its successors will depend on addressing key challenges: improving performance for highly flexible and noncanonical systems, integrating ligand and cofactor data for better interaction modeling, and expanding its applicability to multi-component assemblies. The incorporation of experimental data and interdisciplinary approaches will be critical in overcoming these limitations, ensuring that the new model continues to bridge the gap between computational prediction and biological reality. In conclusion, AlphaFold3 has not only revolutionized structural biology but also laid a robust foundation for future innovation. As researchers build upon its successes and tackle its challenges, the impact of the new algorithm will continue to resonate across biomedical research, enabling deeper insights into the molecular underpinnings of life and driving transformative advancements in science and medicine.

## Figures and Tables

**Table 1:** Applications of AlphaFold3 and novel insights on structural biology and mechanisms.

Case Study	Application of AlphaFold3	Domain	Reference
Evaluate docking fatty acids to FABPs	Predicted binding sites and poses for fatty acids	Drug Discovery/Metabolic Research	[23]
Integrate AF3 with LFIA for pyraclostrobin detection	Modeled antibody–antigen complexes for immunoassay	Environmental Monitoring	[24]
Assess AF3 in predicting RNA structures	Predicted 3D RNA structures	RNA Structure Prediction	[25]
Analyze knotted protein structures	Modeled protein knots and classified knot types	Protein Folding	[26]
Integrate sequence- and structure-based classifications	Modeled protein domains to link Pfam and ECOD	Structural Biology	[27]
Study Zn^2+^-mediated inhibition of caspase-3	Predicted Zn^2+^-binding sites	Computational/Apoptosis/Enzymology	[28]
Assess hookworm tubulin dimerization mutations	Modeled wild-type and mutant tubulin dimers	Computational/Parasitology Experiments	[29]
Enhance AF3 for identifying LC3-interacting regions	Fragment-based modeling for protein–peptide interactions	Method of Autophagy Development	[30]
Model intrinsically disordered proteins (IDPs)	Predicted secondary structure in *α*-synuclein	Computational/Neurodegeneration Experiments	[31]
Explore SARS-CoV-2 M_pro_ dimerization	Predicted protein–protein interaction interfaces	Computational/Virology Experiments	[32]
Model Polo-like kinases and activation mechanisms	Predicted structural variations and conformational changes	Comparative Cancer Biology	[33]
Investigate NLR protein resistosome formation	Predicted structures of NLR hexameric assemblies	Computational/Plant Immunity Experiments	[34]
Study anomalous fluorescence in *α*-helical peptides	Predicted structural motifs influencing fluorescence	Integrative Biophysics	[35]
Characterize cysimiditides and Zn-tetracysteine motifs	Predicted tertiary structures and enzyme interactions	Structural Peptide Biochemistry	[36]
Model human F-ATP synthase structure	Predicted subunit interactions and drug-binding sites	Drug Bioenergetics Discovery	[37]
Study TCR recognition of NRAS cancer neoantigens	Predicted TCR–neoantigen-binding interactions	Computational/Cancer Experimental Immunotherapy	[38]
Model GluN3 NMDA receptor structures	Predicted ligand-binding domains and channel architecture	Neurobiology	[39]
Analyze heme-binding sites in heme proteins	Predicted coordination of heme and protein residues	Structural Biology	[40]
Study impact of N-terminal capping on GeX-2 stability	Predicted structural effects of N-terminal modifications	Peptide Therapeutics	[41]
Develop vaccines using ESAT6/Tb10.4 protein	Predicted immune epitopes of fusion protein	Vaccine Development	[42]
Model Rad52 superfamily DNA repair proteins	Predicted DNA-binding domains and protein interfaces	Genome Stability	[43]
Identify *β*-protein biomarkers for Alzheimer’s	Predicted mutation effects on *β*-protein folding	Neurodegeneration	[44]
Study LuxS enzyme in Klebsiella pneumoniae	Predicted interactions in AI-2 signaling pathway	Microbiology	[45]
Model plastocyanincytochrome f interactions	Predicted interfaces and conformational changes	Photosynthesis	[46]
Binding free energy changes upon mutation	Predicted BFEs in SKEMPI protein complexes	Protein Engineering	[47]
Evaluate chimeric protein structure prediction	Analyzed performance of AF3 for terminal peptide tags	Synthetic Biology	[48]
Characterize MoxR AAA+ ATPase stress resistance	Predicted hexameric assembly and functional regions	Microbial Biology	[49]

**Table 2:** Applications of AlphaFold3 in biomolecular interactions.

Case Study	Application of AlphaFold3	Domain	Reference
Evaluate nanobody–antigen interactions	Predicted nanobody epitopes	Structural Biology	[51]
Evaluate docking capabilities for antibody and nanobody complexes	Predicted antibody–antigen interfaces	Immunology	[52]
Predict RNA–ligand interactions	Modeled RNA–ligand binding interfaces	RNA Structure Prediction	[53]
Evaluate RNA modeling capabilities	Modeled RNA secondary and tertiary structures	RNA Biology	[54]
Predict metal-binding sites in proteins	Identified physiologically relevant metal ion-binding sites	Metalloprotein Modeling	[55]
Analyze repetitive protein sequences	Predicted *β*-solenoid structures	Protein Folding	[56]
Model fold-switching protein conformations	Predicted fold-switched conformations	Structural Biology	[57]
Evaluate robustness with adversarial protein sequences	Predicted high-confidence scores for perturbed sequences	Protein Engineering	[58]
Model human–parasite interactions	Predicted interaction interfaces	Parasitology	[59]
Predict mutational effects on protein–protein interactions	Modeled binding energy changes (∆∆*G*)	Protein–Protein Interactions	[60]
Model PROTAC-mediated protein–protein interfaces	Predicted transient protein interactions	Targeted Protein Degradation	[61]
Enhance epitope prediction for PD-L1:Affibody interactions	Predicted epitope residues	Immunology	[62]
Predict peptide–MHC class II binding cores	Identified pMHC-II binding cores	Immunology	[63]

**Table 3:** Complementary tools that use and extend the application of AlphaFold3.

Tool	Functionality	Reference
AlphaBridge	Analysis of macromolecular complexes, leveraging AlphaFold3’s confidence metrics for efficient validation and visualization.	[65]
AlphaCRV	Ranking of potential protein–protein interactions based on structural and sequence-based clustering, identifying biologically relevant binders by their consistency in structure and topology.	[66]
GalaxySagittarius-AF	Searching for potential drug targets, matching drug-like compounds with predicted protein structures.	[67]
AlphaFLOW-Lit	Capture of protein flexibility and guiding drug design.	[68]

## Data Availability

No new data were created or analyzed in this study. Data sharing is not applicable to this study.
